# Delay and its Related Factors in Seeking Treatment in Patients with Acute Myocardial Infarction

**Published:** 2010

**Authors:** Mohsen Taghaddosi, Mansour Dianati, Javad Fath Gharib Bidgoli, Javad Bahonaran

**Affiliations:** 1 Department of Medical-Surgical Nursing, Kashan University of Medical Sciences, Kashan, Iran; 2 Medical Student, Kashan University of Medical Sciences, Kashan, Iran; 3 Department of Medical Technology, Kashan University of Medical Sciences, Kashan, Iran

**Keywords:** Prehospital delay, Myocardial infarction, Onset-to-door time

## Abstract

**BACKGROUND:**

Early diagnosis and treatment of myocardial infarction can prevent life-threatening complications such as dysrhythmias and death. The aim of this study was to determine the length of delay and its related factors in seeking treatment among a group of patients with myocardial infarction.

**METHODS:**

In a cross-sectional design, all the patients who had referred to a general teaching hospital (Kashan, Iran) for treatment of myocardial infarction from April 2004 to March 2005 were recruited. Demographic characteristics, the amount of delay, and the causes of having delay were recorded.

**RESULTS:**

Two hundred patients were recruited for this study from which 131 (69%) patients had delay in seeking treatment. Factors such as gender, age, economical status, educational level, referring to a general physician before referring to the hospital, the severity of symptoms, residential place (urban vs. rural), and the time of the onset of the symptoms (day vs. night) were determined to be related to having delay. The most important causes of having delay were: "hoping the symptoms to alleviate spontaneously", "attributing the symptoms to other problems other than heart problems", and "disregarding the symptoms".

**CONCLUSION:**

Regarding the most important causes of having delay in this study, the importance of educating people about the symptoms of myocardial infarction and the importance of early referral to the hospitals is clarified.

## Introduction

Coronary artery diseases (CAD) are one of the most prevalent diseases in industrial countries.^1^, ^2^ They are the leading cause of death in 39.4% of cases worldwide.^3^ Approximately 40 to 60 percent of acute myocardial infarction (AMI) deaths usually occur during the first hour after the onset of the AMI symptoms before arriving to the hospital.^4^, ^5^ The morbidity and mortality rate drastically decrease in patients who received therapeutic modalities during the first two hours after the onset of AMI symptoms.^6^, ^7^ Nitrates, beta-blockers, thrombolytics, anti-coagulants, and interventional procedures such as percutaneous transluminal coronary angioplasty and coronary artery bypass grafting are the most important therapeutic modalities for AMI.^5^, ^8^, ^9^ Although such therapies have led to major improvements in patient outcomes, their full potential has not been realized because they are often performed too late.^10^ The quotation "time is muscle" is used to highlight the importance of saving time and starting treatments without delay.^11^, ^12^ Although treatment for AMI should begin within 1 hour of symptom onset, unfortunately the current median time between the onset of AMI symptoms and admission to the hospital is slightly more than 2 hours^13^ and, almost 25% of AMI victims still have a longer than 5 hours delay.^14^ Every 30 minutes of delay increases the 1-year mortality risk by 7.5%.^15^ The period between the onset of symptoms and the decision to call for medical assistance remains the most important cause of total pre-hospital delay.^16^–^18^ Robert reported that, the pre-hospital factors, such as the time between chest pain initiation and deciding to seek treatment, are the biggest source of having delay in Scotland.^8^ AMI patients often use denial during the first hours and even first days after chest pain initiation.^19^ It is an unconscious physiologic response which empowers the patient to encounter and overcome his anxiety and fear. AMI patients often have delay in seeking treatment as a result of denial, relating the symptoms to other than cardiac problems.^17^, ^20^, ^21^ Studies showed that patients usually do actions such as taking a break, using over-the-counter medicines, calling emergency medical services, and consulting a physician for their AMI symptoms.^20^, ^22^, ^23^ Many factors such as being old,^24^ being female,^25^, ^26^ having low socioeconomic status,^27^ and being Black,^28^ clinical factors such as a history of hypertension or diabetes,^25^ or prior history of angina or previous AMI,^27^ and other factors such as consultation with one's spouse, family member or physician^29^ have been associated with longer delay. In Iran, however, there are a few studies on this critical subject area; Soltani reported that the onset-to-door time and the door-to-needle time were 106 and 51 minutes respectively. They also determined that "the home-to-hospital distance", "self therapy" or "using over-the-counter drugs", and "relating the symptoms to other than heart problems", were the main reasons of having delay.^30^ Masoomi and Nikian also found that only "a history of diabetes" and "the severity of chest pain" had relationship with having delay in seeking treatment after chest pain.^31^ They also found that the mean onset-to-door time was around 5 hours. However, these are local rather than national studies and hence can't provide a valid median onset-to-door time for Iranian patients. In the other hand, cultural effects make different behaviors in society and more studies were needed. The aim of this study was to determine the length of delay and the related factors in seeking medical treatment in a group of Kashanian people.

## Materials and Methods

This study with cross-sectional design was done on all patients (200 cases) who were hospitalized and treated due to STEMI (S-T elevation myocardial infarction) in emergency ward and CCU (cardiac care unit) of Kashan Shahid Beheshti Hospital from April 2004 to March 2005. Cases were selected by convenience sampling. Patients admitted after resuscitation from cardiac arrest, diagnosed as AMI after admission for another condition (e1g1 to e. g.) or those with uncertain onset time were excluded. The information about patients was collected by filling out a questionnaire including variants of age, sex, revenue, educational level, cardiac history disease and patients' lab tests. The information about the first place of referral after the pain began, delay cause and the time of starting the pain was gathered. Those who came in less than 2 hours were on time, in 2–4 hours had slight delay, and in 4–8 hours had average delay and more than 8 hours had long delay 2. Then the data were obtained and analyzed by descriptive statistics and chi-square, OR and CI with confidence limit of 95%.

## Results

Among 200 patients, 138 (69%) were men and 65.5% came with delay. The women had more delay and this difference was significant (P=0.029). In-time refer in men was54 (39.1%) and in women was15 (24.2%). By increase of age, the delay increased too; and the difference was meaningful (P=0.0008). Delay in people with low revenue was more than the ones with high revenue, Most of people who had low income delayed and their in-time refer was less than those who had high income (Table 1).

**Table 1:** Abundance distribution in patients with infarction with respect to related factors and in-time refer

Background factors	In time		Referral delay		P value

	n	%	n	%	
Age (year):					< 0.0008
30–45	17	68	8	32	
45–60	19	30.6	43	69.4	
> 60	33	29.2	80	70.8	
Literacy:					< 0.01
Illiterate	25	25.5	73	74.5	
Under diploma	29	39.1	45	60.9	
Above diploma	15	53.5	13	46.5	
Income:					0.067
Low	9	21	34	79	
Moderate	41	36	73	64	
High	19	44.2	24	55.8	
Sex:					
Male	54	39.1	84	60.9	p=0.029 OR=2.014 CI (1.026–3.596)
Female	15	24.2	47	75.8	p=0.878 OR=0.94 CI (0.48–1.17)
Background disease:					
Yes	52	33.7	102	66.3	
No	17	37	29	63	

This study showed that the least rate of the long delay was in patients who came directly to hospital (Table 2 and 3). There was a meaningful relation between the referal condition and the first referral place after MI (P=0.025).

**Table 2:** Abundance distribution in patients with myocardial infarction with respect to the first place of referral after pain and the referral situation

Patients' referral situation	The first refer after pain								
	
	Hospital		Office		City emergency		PHC *		Sum

	n	%	n	%	n	%	n	%	
In time	20	29	6	8.7	30	43.5	13	18.8	69
With slight and moderate delay	19	28	10	15	26	39	12	18	67
With log delay	7	11	17	26	32	50	8	13	64
Sum	46	23	33	16.5	88	44	33	16.5	200
P value	0.025								

* Primary health care

**Table 3:** Abundance distribution in patients with myocardial infarction with respect to patient's transfer to hospital and referral situation

Patient's referral	Quality of transferring to hospital				
	
	Another vehicle		Emergency 115		Sum

	n	%	n	%	
In time	63	91.3	6	8.7	69
With slight and moderate delay	58	86.5	9	13.5	67
With long delay	50	78	14	22	64
Sum	171	85.5	29	14.5	200
P value	0.0933				

The survey results showed that patients who used the vehicles except for emergency ambulances had less delay.

Pain location in 173 cases (86.5%) was chest and left hand which allowed the highest delay (32.4%). The patients with myocardial infarction along with cardiac pain experienced concomitant symptoms such as nausea, vomiting, dyspnea (Table 4).

**Table 4:** Abundance distribution in patients with myocardial infarction with respect to accompanying symptoms and patients' referral situation

Patient's referral	Accompanying symptoms				

	No		Yes		Sum

	n	%	n	%	
In time	8	11.5	61	88.5	69
With slight and moderate delay	8	11.5	59	88.5	67
With long delay	16	25	48	75	64
Sum	32	16	168	84	200
P value	0.0586				

In-time referral in patients who had concomitant symptoms was more than those who didn't have the symptoms (P=0.0586) (Table 4). Among 16 patients who had long delay due to attributing the pain to non-cardiac causes, 14 ones had pain in left chest among whom 9 (64.3%) had concomitant symptoms and 5 (35.7%) didn't. Also from 32 patients who had long delay because of waiting for spontaneous improvement, 27 ones had pain in chest among whom 21 (77.8%) didn't have accompanying symptoms and 6 (22.2%) had. Among 200 under-study patients, 113 ones were inside the city at the time of cardiac symptoms happening which allowed most to refer on-time 37 (37.2%). The most common cause of long delay 32, (42%) was to wait for improvement and in the second place was to attribute the pain to non-cardiac causes 16 (21%). Low educational information in the illiterate people is more common with respect to other causes of long delay (Table 5).

**Table 5:** Abundance distribution in patients with myocardial infarction with respect to education and common causes of delay

Delay reason	Literate		Illiterate		Sum	

	n	%	n	%	n	%
Waiting for spontaneous recovery	20	47.6	12	35.3	32	42.1
Attributing to non-cardiac causes	11	26.2	5	14.7	16	21.1
Not minding the pain	6	14.3	8	23.5	14	18.4
Decrease in educational information	5	11.9	9	26.5	14	18.4
Sum	42	5 5.3	34	44.7	76	100
P value	0.18					

Patients who had chest pain in the night had the longest delay 11 (52.4%); the patients whose pain started early night and early morning were 46.5% and 28.8% respectively. Meanwhile the percent of in-time referral before noon was more than other times of the day. Among 200 under-study patients, 80 ones (40%) informed relatives, and 20 ones (10%) used emergency call 115. Regarding the medication treatment after the start of the pain, from 64 patients who came with long delay, 43.8% (28 ones) had taken no medication measure and 25% (16 ones) had taken sublingual TNG.

## Discussion

In this study, from 200 patients, two thirds were men. Women had more delay comparing to men and also most of them had long delay (45.1%). According to Dracup, between 1/4–1/2 patients with MI had delay more than 6 hours from the beginning of the symptoms.^32^ For every 30 minutes delay in sending the patients to hospital, the probability of decrease in human life span increases by 7.5% for one year; and 30–40% of the patients who had delay were looking for help for more than 6 hours, and doing nursing actions reduced the delay time from the average of 5.7 hours to 5.5 hours.^18^ Gilber has mentioned 110 minutes as the average time of the onset of symptoms till arriving to the hospital. African women had the most delay.^20^ In a study in Scotland in 2000, Robert showed that women had the most delay in referring to hospital. Perhaps the reason for these results is the high threshold of pain tolerance in women or more common rate of MI in men, and that women don't attribute chest pain to heart and its related diseases and so they don't act to reduce it. On the other hand, the women are influenced with heart attacks in older ages, so their sense of pain may decrease with age; and this pain may become more tolerable.^33^

The findings of the table 1 show that with advancing age, the rate of delay has generally increased. The people older than 60 years old often came with slight and long delay in proportion to the previous groups. In a study by Cramlish in 2000 on patients with acute MI it was showed that as age increases, the rate of delay also increases.^4^ Boresma mentioned that the most common cause of delay in patients with infarction was increasing age (more than 45 years).^34^ The results of these findings may be due to high pain threshold in older people; or the increase in personal knowledge and experience has caused the delay for referring to hospital.^34^ The rate of in-time referal in patients has a reverse relation with their situation of revenue; namely the patients whose income is lower have more delay and have less in-time referal in comparison with others. In a study by Robert in 2000 patients who had low income had also the longest delay.^8^ Also Lisa described that one of the reasons for delay was low life income.^35^ The above findings have been coincident. Perhaps high treatment expense and patients' low income have been the reasons for the delay in patients with MI. Illiterate patients had more delay compared to those with educated ones and the abundance of long delay in this group was more; and this finding is in accordance with Lisa's study in 2000,^35^ but Rosenfeld (2001) did not introduce low education as the reason for delay but remarks the low knowledge and information from cardiac disease and the complications due to lack of in-time treatment as the most important cause. The longest delay has been in patients who had a positive history of underlying diseases (HTN, DM)^1^ and the reason for in-time refer in patients with infarction along with diabetes is probably because of physician's education based on the possibility of lack of pain and taking every chest pain into consideration and the necessity of in-time referral which needs more survey. Also 84 people who had a hospitalization history because of heart problems did not have such differences with the ones who never had the former history. The reasons for delay are divided into two parts: the background causes (female sex or the older ages) and clinical (DM and Angina history) and the environmental factors (physician's consult with one of the members in the family at the time of the event) and the emotional reactions (anxiety and bothering others, fear from outcomes)^18^ and in a study by Novis (1998) on the patients with MI, the ones with a positive history of HTN and previous MI, had more delay.^16^ This point is of high importance because some attributed these symptoms to their previous disorders with respect to not having previous experiences, but the patients with cardiac disease often have personal experience and this is a factor that remarks the in-time refer and the necessity of attention to the ones who have risk factors like DM and HTN so that they can have enough education. On the other hand, some diseases such as diabetes increase the pain threshold.^36^ The findings show that the longest delay was among the patients who had come directly to the general practitioner and those who had come to the city emergency or public health service. In a study in Scotland in 2000, one of the most common causes of pre-hospital delay was the general practitioners because of insufficient experience and wrong diagnosis which confirms the present findings.^8^ In this study the delay was on the part of the patients who used emergency service 115. In a study by Novis in 1998, the ones who called ambulances had a mean delay about 1/3 an hour less than the ones who had not called the ambulances directly.^16^ The difference between these two studies is perhaps due to the long time of decision-making to use emergency service 115, the patient's last action after not being well, or pain and/or the decreased speed of transferring the patient by emergency call 115. In-time referal was mostly in patients who had pain in chest and left hand; perhaps the previous experience about similar non-cardiac pain in chest and after that or low severity of the pain have been the reasons for this delay which is considerable. The patients who had experienced symptoms like nausea, vomiting, sweating and dyspnea and etc. with cardiac pain came sooner than the group which did not have the accompanying symptoms. Existence of concomitant symptoms can reduce patient's pain and tolerance threshold and can make the pain ambiguous from the viewpoint of the patient.^21^ The ones who have been inside the city at the time of the pain had more in-time refer comparing to those from outside the city. Lack of access to transfer means and rural culture can be the reasons for delay in referring of patients. The most common reason for long delay in an order of abundance are as follows respectively: waiting for spontaneous improvement, attributing to non-cardiac causes and not minding the pain and low educational information. Robin's study (1999) showed that many of patients wait for spontaneous recovery for 24 hours after the beginning of the symptoms and 60% of cardiac mortality has been before getting to hospital^19^ and low medical information.^36^ Low medical knowledge in illiterate people about ischemic heart disease has been the most common cause of delay with respect to the highly educated people, and this finding is similar to the other studies.^18^, ^23^, ^28^ But Gilber has stated that the common factors in patients who delayed were being old, having low income, DM and relating the pain to non-cardiac causes and the intermittency of the symptoms.^20^

The difference between these two groups is probably related to the patient's anticipation for recovery or lack of an experienced physician and lack of sufficient equipment during night especially personal transportation. As far as Dracup showed, the mortality rate of patients with MI in hospital had a meaningful relation with the delay of treatment, and the reasons were lack of intervention such as patient and his family's education about the causes and complications of MI at the beginning of the treatment.^20^, ^33^ Luepker reminded alarming program of the cardiac attacks outbreak regularly and widespread comprehensive education for decrease of delay from the beginning of the symptoms till presence in hospital.^37^ But Dracup believes that instead of using general education, a face-to-face education should be done by a nurse so that these actions can reduce the main emotional, social and perceptive obstacles which were known as the progressive factors of delay before hospital in last studies.^3^ The ones who used sublingual tablets to relieve pain had 25% long delay. Also the patients who took pain killer had the least percent of in-time refer. This shows that the lack of cognition of cardiac pain importance and its symptoms and waiting for recovery caused delay in in-time refer.^18^, ^24^

## Conclusion

Causes of delay and lack of in-time treatment in more than half of the patients include waiting for spontaneous recovery, attributing the chest pain to non-cardiac causes, not minding the pain and decreased educational information and they have many complications for patients with MI. Regarding referral delay and high prevalence of this disease in our country and on the other hand, not knowing its essence for patients, it needs to survey on methods of encouraging the patients with MI for in-time refer to physicians along with suitable educations about the symptoms.
